# Vehicles of Malaria

**Published:** 1879-12

**Authors:** 


### VEHICLES OF MALARIA.

Ague is commonly supposed to be due to the entrance of a miasmatic organism into the system. But no microscope has ever seen this organism, neither can we account for the intermittance of the ague paroxysms, nor can we say for certain through what way it finds its way into the system.

The majority of writers hold the opinion that *the air of marshes* is the sole cause of intermittent fever. But there exists strong evidence going to show that water, too, is a carrier of the poison. Take for instance two or three cases cited in the *Lancet.* First, the case recorded by Boudin, of three vessels sailing from Algiers to Marseilles, conveying 800 soldiers, who on shore had all been exposed to the same atmospheric condition. Two of these vessels were supplied with good water, but the third with water from a marsh. The two former arrived at Marseilles without a sick man, but the third ship lost thirteen men and had 120 sick, nine-tenths of whom were affected with malaria. Again, there is the outbreak of ague at Tilbury Fort, cited in Parkes' *Hygiene,* where thirty-four men out of a garrison of 103 were seized with ague, while the people at the railway station, and the coast guard men and their families just outside the fort, entirely escaped. The troops had been supplied with water stored in tanks, collected from the rain water of the roofs, while the people outside obtained theirs from a spring, the atmospheric condition in both cases being identical.—*Popular Science Monthly.*

